# Comprehensive Assessment of Genetic Sequence Variants in the Antioxidant ‘Master Regulator’ Nrf2 in Idiopathic Parkinson’s Disease

**DOI:** 10.1371/journal.pone.0128030

**Published:** 2015-05-26

**Authors:** Michael Todorovic, Jeremy R. B. Newman, Jianguo Shan, Steven Bentley, Stephen A. Wood, Peter A. Silburn, George D. Mellick

**Affiliations:** 1 Eskitis Institute for Drug Discovery, Griffith University, Nathan, Queensland, Australia; 2 Asia-Pacific Centre for Neuromodulation, UQCCR, University of Queensland, Herston, Queensland, Australia; Mayo Clinic, UNITED STATES

## Abstract

Parkinson’s disease (PD) is a complex neurodegenerative disorder influenced by a combination of genetic and environmental factors. The molecular mechanisms that underlie PD are unknown; however, oxidative stress and impairment of antioxidant defence mechanisms have been implicated as major contributors to disease pathogenesis. Previously, we have reported a PD patient-derived cellular model generated from biopsies of the olfactory mucosa, termed hONS cells, in which the NRF2-mediated antioxidant response pathway genes were among the most differentially-expressed. To date, few studies have examined the role of the NRF2 encoding gene, *NFE2L2*, and PD. In this study, we comprehensibly assessed whether rare and common *NFE2L2* genetic variations modify susceptibility to PD using a large Australian case-control sample (PD=1338, controls=1379). We employed a haplotype-tagging approach that identified an association with the tagging SNP rs2364725 and PD (OR = 0.849 (0.760-0.948), *P* = 0.004). Further genetic screening in hONS cell lines produced no obvious pathogenic variants in the coding regions of *NFE2L2*. Finally, we investigated the relationship between xenobiotic exposures and NRF2 function, through gene-environment interactions, between *NFE2L2* SNPs and smoking or pesticide exposure. Our results demonstrated a significant interaction between rs2706110 and pesticide exposure (OR = 0.597 (0.393-0.900), *P* = 0.014). In addition, we were able to identify some age-at-onset modifying SNPs and replicate an ‘early-onset’ haplotype that contains a previously identified ‘functional promoter’ SNP (rs6721961). Our results suggest a role of *NFE2L2* genetic variants in modifying PD susceptibility and onset. Our findings also support the utility of testing gene-environment interactions in genetic studies of PD.

## Introduction

Parkinson’s disease (PD) is a complex neurodegenerative disorder influenced by a combination of genetic and environmental factors. The molecular mechanisms that underlie neurodegeneration in PD are unknown; however, recent studies have highlighted the significant role that oxidative stress and impairment of antioxidant defence mechanisms play as major contributors to disease pathogenesis [1–3].

Previously, we have reported a PD patient-derived cellular model generated from biopsies of the olfactory mucosa (termed human olfactory neurosphere-derived (hONS) cells) that demonstrate disease-specific differences in gene expression, protein function and metabolic activity, when compared to healthy controls [4]. Subsequent bioinformatics pathway analysis highlighted the “NRF2-mediated antioxidant response” pathway as one of the most altered in PD hONS cells.

Nuclear factor erythroid-2-related factor 2 (protein: NRF2; gene: *NFE2L2*) is a transcription factor in the phase II antioxidant and xenobiotic response pathway and is termed a ‘master regulator’ of expression for many antioxidant and detoxification pathway genes [5]. At basal levels, NRF2 is constitutively degraded in the cytoplasm by its antagonist, KEAP1 ('Kelch-like erythroid-cell-derived protein with CNC homology (ECH)-associated protein 1'). KEAP1 forms a complex with CUL3-RBX1 and regulates NRF2 through targeted ubiquitination and subsequent degradation. The mode of action for NRF2 begins upon exposure to oxidative stress, xenobiotics, or electrophilic compounds. This causes the modification of cysteine-151 of KEAP1 and subsequent stabilisation and translocation of NRF2 to the nucleus where it binds to the antioxidant response element (ARE) of its target genes [5, 6].

Recent *in vitro* and *in vivo* studies have demonstrated a potential neuroprotective role of NRF2, attenuating neurotoxicity in 6-hydroxydopamine, hydrogen peroxide, and 1-methyl-4-phenyl-1,2,3,6-tetrahydropyridine (MPTP) induced models of PD [7–9]. NRF2 activity and expression also significantly fall with age, the most common predisposing factor for PD [10]. Subtle accumulative changes in antioxidant response mechanisms may contribute to the oxidative damage previously identified in the post-mortem midbrain of PD patients [11]. In addition, other molecules directly or indirectly regulated by NRF2 have been strongly linked with PD; these include glutathione [12], heme oxygenase-1 (HO-1) [13, 14], and NAD(P)H:quinone oxidoreductase 1 (NQO1) [15]. Functional experiments performed using our PD hONS cell lines have further demonstrated reductions in associated metabolic function, including reduced levels of glutathione, suggesting deficiencies in NRF2 function [16]. Moreover, these deficiencies were ameliorated after induction of the NRF2-mediated antioxidant response pathway with l-sulforaphane, an NRF2 activating agent.

Despite this putative neuroprotective role of NRF2, there have been few studies examining associations between *NFE2L2* and PD. A candidate gene approach was undertaken in a Taiwanese cohort and two independent European (Swedish and Polish) case-control groups [17, 18]. Of the 11 single nucleotide polymorphisms (SNPs) investigated, none were significantly associated with PD in either cohort. However, associations between a specific haplotype and PD were reported in both European case-control groups. Interestingly, this haplotype includes a previously described ‘functional variant’ of the promoter [19] and was associated with delayed age at onset (AAO) in the Swedish material and reduced risk of PD in the Polish material [17]. Both case-control groups used in this study were modestly sized and not adequately powered to unequivocally detect the reported associations.

We decided to investigate whether genetic variations in and around *NFE2L2* modify susceptibility to PD using a large case-control sample recruited via the Queensland Parkinson’s Project. We employed a haplotype-tagging approach to comprehensively determine if common SNPs around *NFE2L2* are associated with PD. In addition, we also screened the coding regions of *NFE2L2* to determine if rare coding variants could be responsible for the functional alterations observed in our PD hONS cell lines. Finally, because of the relationship between xenobiotic exposures and NRF2 function, we investigated possible gene-environment interactions between *NFE2L2* SNPs and smoking or pesticide exposure.

## Methods

### Case-control ascertainment

Patients with neurologist diagnosed idiopathic Parkinson’s disease (n = 1338) and neurologically normal, healthy, community-dwelling control subjects (n = 1379) were recruited as a part of the Queensland Parkinson’s Project, a collaborative research study register of over 4000 community-dwelling Queenslanders, recruited since 2005, who have agreed to participate in research into Parkinson's disease and related disorders [20]. All subjects were of European Caucasian background and were recruited by experienced neurologists from specialist movement disorder clinics in Brisbane, Australia and the Australian electoral roll, and detailed epidemiological data has been obtained from these individuals. Study participants provided blood samples from which DNA was extracted using standard methods. A subset of the sample (67 individuals) also provided olfactory mucosa biopsies [4]. Study demographic data is presented in Table 1.

**Table 1:** Study demographics.

Factor	Control	PD	Comment ^a^
All individuals	1379	1338	
Mean age ± SD	68.84±10.77	71.14±10.36	*P*<0.001 ^b^
Mean age at onset ± SD	n/a	59.28±11.44	
Male/Female	634/745	837/501	PD: OR = 0.51 (0.44–0.60) *P*<0.001
Cigarette smoking ^c^ Median Packyears (Range)	19.65 (0.1–176.25)	14 (0.01–212.5)	Range: 0.01–212.50 *P* = 0.001 ^b^
Cigarette smoking ^c^ Mean rank	540.46	476.87	
Pack years (≥ 17 median) ^d^	308/973 (31.57%)	206/1007 (20.46%)	PD: OR = 0.65 (0.53–0.79) *P* = <0.001
Pesticide exposure (>26 days life exposure) ^e^	141/1146 (12.30%)	231/992 (23.29%)	PD: OR = 1.89 (1.51–2.37) *P*<0.001

^a^ Odds ratio (95% CI) calculated using binary logistic regression

^b^ Wilcoxon rank sum test

^c^ pack years equivalent to packs per day multiplied by years smoked

^d^ pack years dichotomised according to median <17 & ≥ 17 pack years (zero pack years removed)

^e^ Pesticide exposure is equivalent to exposure of herbicides, pesticides, or fungicides at least once weekly for a period of six months.

### Ethics Statement

Written informed consent was obtained from all participating subjects in this study via a signed subject consent form, which is held in hard copy by the authors. The study and the consent procedures were approved by the Human Research Ethics Committee of Griffith University under clearance numbers: ESK/04/11/HREC “The Queensland Parkinson's Project—uncovering genetic risk factors for Parkinson's Disease"; and ESK/04/07/HREC “Olfactory biopsies in Parkinson’s disease and related disorders”. All participants had the capacity to provide individual consent at the time of recruitment.

### High Resolution Melt analysis

High Resolution Melt (HRM) analysis was employed to screen the *NFE2L2* coding regions, including the 5’ and 3’ UTR of DNA derived from 67 PD and control hONS cell lines. Assays were designed to amplify sequences no more than 200bp long, with final amplicon lengths between 145 and 183bp (S1 Table). *NFE2L2* exons were sequenced in a randomly selected control to serve as reference samples. HRM analysis was performed using the Corbett Rotor-Gene 6000 Real-Time PCR system (Qiagen, Doncaster, Australia) and the Sensimix HRM Master Mix (Bioline, Alexandria, Australia). Assays were carried out as per manufacturer’s instructions and standard laboratory protocols.

### SNP and haplotype association analysis

We utilised linkage disequilibrium-based mapping approaches to “tag” all known common variants ± 5kb (minor allele frequencies > 10%, r^2^ > 0.90) of *NFE2L2* (S1 Fig). *NFE2L2* SNPs previously genotyped in published case-control association studies of PD [17] were also genotyped here and, where possible, were used to complement or substitute tagging SNPs derived from HapMap data. This approach produced 11 tagging SNPs and was derived from the HapMap project CEU population (data rel 28/phase II+III, August10, on NCBI B36 Assembly). Assays for the 11 tagging SNPs—rs13035806, rs2706110, rs10183914, rs2001350, rs1806649, rs2364722, rs2886161, rs2364725, rs7557529, rs16865105 and rs6726395 were designed and executed using the MassARRAY genotyping platform (Sequenom, San Diego, USA).

In total, 11 tagging SNPs were screened in 1338 PD and 1379 control subjects. Three *NFE2L2* promoter SNPs (rs6721961, rs6706649, rs35652124) previously genotyped in published age-of-onset studies of PD [17] were also genotyped in 502 PD and 189 control subjects. SNP genotypes were analysed in PLINK version 1.07 (http://pngu.mgh.harvard.edu/~purcell/plink/). Hardy-Weinberg equilibrium was calculated for all SNPs to assess genotyping reliability. Odds ratios (ORs) for genotype and haplotype associations (additive model) were calculated using logistic regression with adjustment for age and gender. Testing for multiple hypotheses was adjusted using a strict bonferroni correction. This study has 90% power to detect ORs of at least 1.41. *NFE2L2* haplotype windows were phased to include haplotypes >1% in order to confidently replicate previously published age-at-onset (AAO) haplotype associations. AAO is defined as patient-reported ‘age at first primary motor symptom’. Associations between individual SNPs and AAO were evaluated using linear regression models adjusted for gender. P-values were adjusted for multiple hypotheses testing using a strict Bonferroni correction. An overview of SNPs and haplotype definitions can be found on Table 2.

**Table 2:** Overview of SNPs and haplotype definitions.

*NFE2L2* SNP	SNP identifier	Chromosome 2 position (bp)	Major allele	Minor allele	MAF
1	rs13035806	177800068	G	A	10.4%
2	rs2706110	177800408	G	A	17.7%
3	rs10183914	177805912	C	T	34.6%
4	rs2001350	177808671	A	G	9.0%
5	rs6726395	177811475	G	A	43.6%
6	rs1806649	177826398	C	T	24.8%
7	rs2364722	177833033	A	G	34.0%
8	rs2886161	177836085	T	C	33.9%
9	rs6721961	177838283	G	T	9.8%
10	rs6706649	177838317	C	T	13.2%
11	rs35652124	177838319	T	C	33.5%
12	rs2364725	177841234	T	G	45.7%
13	rs7557529	177843343	T	C	46.0%
14	rs16865105	177844875	A	C	18.5%
**Haplotype block**	**Block identifier**	**Comprising *NFE2L2* SNPs**			
von Otter, *et al* ^a^	“VO”	3, 4, 6, 8, 13			
'Tagging'	“T”	1, 2, 3, 4, 5, 6, 7, 8, 12, 13, 14			
Promoter	“P”	9, 10, 11			
von Otter and Promoter	“VOP”	3, 4, 6, 8, 9, 10, 11, 13			
Tagging and Promoter	“TP'”	1, 2, 3, 4, 5, 6, 7, 8, 9, 10, 11, 12, 13, 14			

MAF = minor allele frequency

^a^ Haplotype block is identical to the haplotype block defined as “Tag SNPs 2–6” in von Otter, et al.

### Gene-environment interaction analysis

Gene-environment interactions were tested using an additive model for *NFE2L2* SNPs by the interactive terms: smoking and pesticide exposure. Smoking terms were dichotomised according to median pack years (<17 pk/yrs & ≥17 pk/yrs) and regular pesticide exposure denote a self-reported exposure to herbicides, insecticides or fungicides at least once weekly for a period of six months or more across their life [21]. Pesticide exposure was dichotomised according to regular pesticide exposure and non-regular pesticide exposure. Representative groups were created to reflect the combined effects on risk of PD: (1) common homozygote, non-exposed, (2) common homozygote, exposed, (3) heterozygote, non-exposed, (4) heterozygote, exposed, (5) alternate homozygote, non-exposed and (6) alternate homozygote, exposed. The common homozygote, non-exposed (1) group served as the reference in an additive interactive model. Odds ratios were calculated using binary logistic regression with adjustment for age and gender.

### Exploring AAO effects using Moving Average Plots

Moving average plots (MAPs) of the differences in allele frequency between cases and controls can provide useful information about the true nature of the impact of genotype on the AAO of disease in case control studies [22]. MAPs graphically portray how the differences in allele frequency, between cases and controls, move with the age of the participants in each group. Such an analysis may provide a means to distinguish between risk factors and AAO modifiers. To do this, we ranked all cases and controls for age, and grouped the participants into 10 percentile ranges. We then plotted the frequency distribution of cases and controls as a function of age. MAPs were constructed for all SNPs. SNPs with significant interactive effects and associations are presented.

### Cell culture

hONS cell lines were derived from biopsies of the olfactory mucosa from donor subjects and established as previously described [4]. Aliquots of hONS cell lines from PD patients and healthy controls were grown in Dulbecco’s modified eagles medium/ham’s F-12 (Gibco, Invitrogen) containing 10% foetal calf serum (Gibco, Invitrogen) and incubated at 37°C with 5% CO_2_.

### Gene expression

Genome-wide expression data previously generated for investigating transcriptional differences between PD- and control-derived hONS cell lines was obtained and processed as previously described [4]. Briefly, total RNA was purified from 56 hONS cell lines (30 PDs & 26 controls) and hybridized to Illumina Human-Ref8 v3 BeadChip arrays (Illumina, Inc.). The raw data was summarized using BeadStudio (Illumina, Inc.), and quantile-normalized using the R/Bioconductor lumi package (http://www.bioconductor.org/packages/2.0/bioc/vignettes/lumi/inst/doc/lumi.pdf).

We next conducted an analysis to determine whether the normalized gene expression of *NFE2L2*, *KEAP1*, and *NQO1* was dependent on genotypes at the *NFE2L2* tagging and promoter SNPs. As the gene-expression data was normally distributed for *KEAP1* and *NQO1*, ANOVA tests were performed. *NFE2L2* gene-expression data was not normally distributed; therefore a Kruskal Wallis test was performed to look for genotype dependant differences.

Genotypes were dichotomised according to possession of ‘major allele’ (G/G) and ‘minor allele’ (G/T or T/T).

### Cell viability assays

CyQUANT Cell Proliferation Assays (Life Technologies) were used to determine the cellular response of hONS cell lines treated with 50nM rotenone or DMSO for 24, 48, 72, 96 and 120 hours. CyQUANT Cell Proliferation Assays measure the cellular DNA content via fluorescent dye incorporation. The fluorescence intensity of each sample was measured using the Synergy2 plate reader, set with an excitation ~485nm and emission detection at ~530nm. T-tests were performed using normalised cell viability data and genotype data of previously described *NFE2L2* tagging and promoter SNPs.

## Results

### High resolution melt analysis

Thirty-two assays were designed that spanned the entirety of the protein-coding regions of *NFE2L2*. Unique melt curve profiles were detected in two of 67 DNA samples from hONS cell lines (S2 Fig). Sequencing confirmed the presence of a novel variant in exon 5 (c.1230T>C; p.S410S) in one PD cell line and identified a rare SNP rs34154613 (c.802G>A; p.V268M) in a control cell line. *In silico* analysis with software: PMUT, PolyPhen and SIFT suggested that neither variant is likely to be pathogenic. No additional variants were detected in other *NFE2L2* exons.

### Genotype associations

Significant associations were observed between two *NFE2L2* tagging SNPs and PD (Table 3). The frequency of the alternative alleles of rs2364725 and rs7557529 were significantly higher in controls, although we noted that these SNPs were highly correlated (r^2^ = 0.98; S1 Fig). In addition, the *P*-value for the association of rs2364725 with PD remained statistically significant following a Bonferroni correction for multiple hypothesis testing (*P*<0.05). All genotyping data in the control subjects conformed to Hardy-Weinberg equilibrium.

**Table 3:** Genotyping associations of SNPs and Parkinson’s disease.

SNP	Alleles ^a^	Control	PD	OR ^b^ (95% CI)	*P* ^c^
rs13035806	G/A	1039/287/23	1047/234/23	0.852 (0.718–1.012)	0.068
rs2706110	G/A	860/427/53	874/372/50	0.892 (0.775–1.026)	0.109
rs10183914	C/T	547/615/182	556/573/161	0.911 (0.811–1.023)	0.114
rs2001350	A/G	1121/212/15	1077/212/10	0.978 (0.807–1.185)	0.817
rs6726395	G/A	402/650/302	407/632/266	0.921 (0.825–1.028)	0.141
rs1806649	C/T	738/509/92	727/476/96	0.979 (0.864–1.110)	0.744
rs2364722	A/G	618/570/153	567/561/165	1.114 (0.992–1.251)	0.069
rs2886161	T/C	628/560/152	580/555/158	1.100 (0.980–1.236)	0.107
rs2364725	T/G	367/663/323	404/631/268	0.849 (0.760–0.948)	0.004^d^
rs7557529	T/C	347/653/317	367/634/262	0.869 (0.776–0.973)	0.015
rs16865105	A/C	919/369/56	889/361/58	1.024 (0.892–1.177)	0.734

^a^ Major/minor allele

^b^ Odds ratio

^c^ adjusted for age and gender

^d^ Bonferroni corrected *P*<0.05.

### Moving Average Plots

The MAPs for image A in S3 Fig is considerably lower for patients throughout the age ranges, with few overlapping age groups; this trend is suggestive of a risk association effect. Image B in S3 Fig depicts an intersection of slopes of patients with controls at the 80^th^ percentile, showing enrichment in the later ages for cases and depletion in the earlier ages, indicating an AAO effect. Conversely, image C in S3 Fig shows allele frequency enrichment of cases in the earlier age percentiles and depletion in the later ages. An earlier AAO effect is suggested in image D in S3 Fig with a higher frequency in patients in the earlier age ranges and progressively decreasing. In image E in S3 Fig, MAPs highlight a risk association with a reduced frequency in patients across age groups, most pronounced in the 20^th^–70^th^ age percentiles.

### Age-at-onset SNP effects

AAO data was generated for the 11 tagging SNPs and 3 promoter SNPs (S2 Table). Possession of an alternate allele for rs10183914 is associated with a later AAO of 2.6 years (*P*<0.001, 95% CI = 1.6–3.6). In addition, rs6726395 corresponds with a later AAO of 1.9 years (*P* = 0.002, 95% CI = 0.95–2.9), and rs1806649 with an AAO of 2.4 years later (*P*<0.001, 95% CI = 1.3–3.5). Conversely, rs35652124 presents with an earlier AAO of 2.2 years (95% CI = -3.9- -0.8), however this SNP did not survive testing for multiple hypotheses. There is a modest delayed AAO effect of 1.6 years associated with rs7557529 (*P* = 0.024, 95% CI = 0.6–2.5).

### Gene-environment interaction analysis

In our QPP study sample, PD cases were more likely to be males and reported fewer pack-years of cigarette smoking compared to controls. The cases were also more likely to have reported regular exposure to pesticides (Table 1). Given the role of NRF2 in antioxidant response, we stratified our study sample based on cigarette smoking and regular pesticide exposure to look for interactive effects. No significant interactions were observed for SNPs rs2364725 (which showed a main effect on disease status) or rs10183914, rs35652124, rs6726395, and rs1806649 (which showed main effects on AAO) (S3 Table). However, a significant additive interaction was observed between rs2706110 and pesticide exposure (Table 4), this effect did not survive multiple hypotheses testing. Additionally, no interactive effect was observed for the previously described ‘functional promoter SNP’ rs6721961 (S3 Table).

**Table 4:** Gene-environment interactions.

SNP	Alleles ^a^	OR ^b^ (95% CI)	*P* ^c^	Bonferroni corrected *P*
**Main Effects (without interaction)**				
rs2706110	G/G	ref	-	
	G/A	0.826 (0.689–0.992)	0.041	0.547
	A/A	0.920 (0.598–1.417)	0.706	1
rs2706110 (additive model)		0.892 (0.775–1.026)	0.109	1
Smoking ^d^		0.514 (0.416–0.637)	<0.005	<0.005
Pesticide exposure ^e^		1.539 (1.208–1.960)	<0.005	0.007
**Final Interaction model (additive genetic model)**				
rs2706110		0.943 (0.790–1.125)	0.512	1
Smoking		0.506 (0.393–0.650)	<0.005	<0.005
Pesticide		1.898 (1.410–2.555)	<0.005	<0.005
Smoking ^d^ * rs2706110		1.045 (0.720–1.518)	0.815	1
Pesticide ^e^ * rs2706110		0.597 (0.393–0.900)	0.014	0.193
Pesticide ^e^ * G/G		ref	-	
Pesticide ^e^ * G/A		0.663 (0.393–1.118)	0.124	1
Pesticide ^e^ * A/A		0.265 (0.085–0.819)	0.021	0.294

^a^ major/minor allele

^b^ odds ratio

^c^ adjusted for age and gender

^d^ ≥ 17 pack years (median)

^e^ lifetime exposure to pesticide >26 days.

### Effects of genotype on gene expression in hONS cells

No differences were observed in normalised expression levels of *NFE2L2* between SNPs and common genotypes in our 52 hONS cell lines (image A in S4 Fig). Relative *KEAP1* levels were significantly different (P<0.05) for carriers of three SNPs (image B in S4 Fig). Individuals in possession of the alternate allele rs2706110 and rs13035806 showed significantly reduced *KEAP1* expression relative to wildtype. Conversely, rs6706649 shows a relative increase in expression levels of *KEAP1*.

A significantly higher level of *NQO1* expression was observed in cell lines carrying rs6721961, relative to wildtype (image C in S4 Fig).

### Effects of genotype on cell viability in rotenone exposed hONS cells

S5 Fig shows relative cell numbers for rotenone (50nM) treated time course of hONS cell lines on an allele specific background. Statistically significant differences in cell number, measured at 120 hours post-rotenone treatment, were observed in hONS cell lines carrying rs13035806. At 96 hours and 120 hours, cell lines carrying rs6721961 had a significantly higher cell number compared to those with the common allele.

### Haplotype associations

Haplotype definitions were retained from a previous study [17] to investigate possible influence of susceptibility of PD within our sample. We also examined associations between haplotypes comprised of all SNPs and PD for completeness (Table 5). Our 11 tagging SNPs produced nine haplotypes, of which the haplotype designated ‘T1’ was modestly associated with PD (OR = 1.167 (1.03–1.32), *P* = 0.0149), although this did not survive a correction for multiple hypothesis testing.

**Table 5:** Haplotype frequencies and associations in PD cases and control.

		Australian cohort (current study)				Swedish cohort (von Otter *et al*.)				Polish cohort (von Otter *et al*.)			
Haplotype	Alleles	OR (95% CI)	Control (%)	PD (%)	*P*	OR (95% CI)	Control (%)	PD (%)	*P*	OR (95% CI)	Control (%)	PD (%)	*P*
VO1	CACCT	1.1 (1.0–1.3)	31.6	33.4	0.06	N/A	30.6	26.2	NS	N/A	28.2	33.9	NS
VO2	TATTC	1.0 (0.8–1.1)	23.6	23.1	0.55	0.9 (0.6–1.3)	26.7	21.5	0.45	0.6 (0.4–0.9)	27.7	18.4	0.01
VO3	CACTT	1.1 (0.9–1.2)	18.7	19.6	0.46	N/A	22.0	22.2	NS	N/A	20.5	20.5	NS
VO4	TACTC	0.9 (0.7–1.0)	12.4	11.0	0.13	N/A	9.1	10.2	NS	N/A	7.1	9.9	NS
VO5	CGCTC	1.0 (0.8–1.2)	8.4	8.4	0.86	2.4 (1.2–4.5)	5.5	10.9	0.01	0.9 (0.5–1.5)	9.6	9.7	0.70
VO6	CACTC	0.8 (0.6–1.1)	3.8	3.1	0.14	N/A	N/A	N/A	N/A	N/A	N/A	N/A	N/A
VO7	CATTC	0.9 (0.6–1.6)	1.5	1.4	0.8.	3.7 (1.3–10.6)	1.7	5.4	0.01	0.5 (0.2–1.6)	2.5	1.4	0.24
T1	GGCAGCGCTTA	1.2 (1.0–1.3)	30.8	33.3	0.01 ^a^								
T2	GGTAATATGCA	1.0 (0.8–1.1)	24.6	23.9	0.67								
T3	GGCAGCATTTC	1.0 (0.9–1.2)	17.4	17.7	0.69								
T4	AATAACATGCA	0.9 (0.7–1.0)	11.8	10.2	0.11								
T5	GACGACATGCA	1.0 (0.8–1.2)	7.5	7.3	0.76								
T6	GGCAGCATTTA	1.1 (0.8–1.6)	2.4	2.7	0.53								
T7	GGCAGCATGCA	0.9 (0.6–1.4)	2.3	2.3	0.72								
T8	GGCAGCGCTTC	0.7 (0.4–1.2)	1.7	1.2	0.26								
T9	GGCGACATGCA	1.0 (0.6–1.8)	1.3	1.3	0.87								

N/A = Data not provided

^a^ Bonferroni corrected *P* = 0.164

VO = ‘Von Otter Haplotype’ (rs10183914, rs2001350, rs1806649, rs2886161, rs7557529)

T = ‘Tagging Haplotype’ (rs13035806, rs2706110, rs10183914, rs2001350, rs6726395, rs1806649, rs2364722, rs2886161, rs2364725, rs7557529, rs1685105)

NS = not significant (>0.05).

### Haplotype age-of-onset analysis

**Fig 1:**
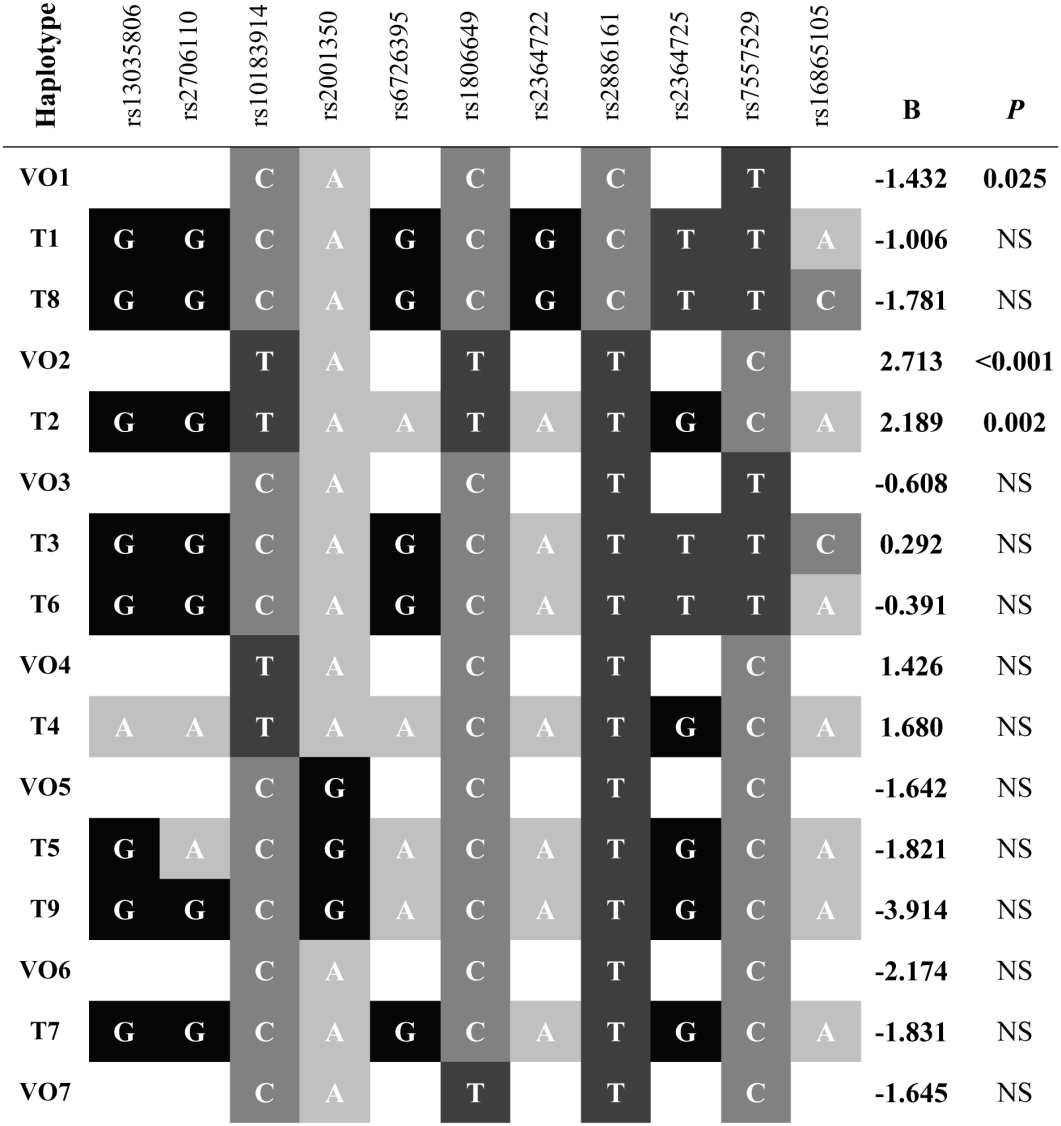
Haplotype construction map (VO1-VO7 & T1–T7) and associated AAO effects. NS = not significant (>0.05).

Our 11 tagging SNPs generated 9 haplotypes, of which, T2 was associated with a delayed AAO (*P* = 0.002, 2.2 years/allele) (Fig 1).

Four phased haplotypes were generated from the three promoter SNPs (S4 Table). We found that haplotypes P2 and P3 were significantly associated with differences in AAO. A reduction of 4.6 years per copy was observed with haplotype P2, and conversely haplotype P3 was associated with an increase in AAO of 3.4 years per copy.

## Discussion

Previously, we have reported disease-related differences in the NRF2-mediated antioxidant response pathway in hONS cell lines derived from patients with PD [16]. In the current study we attempted to comprehensively investigate whether coding region genetic variations in the NRF2 encoding gene could account for these differences. While we observed two rare *NFE2L2* variants of unknown pathogenicity in 67 hONS cell lines, our results indicate that the NRF2-pathway differences previously observed in hONS cell lines are not likely driven by these variants. We also tested for genetic associations between SNPs that comprehensively tag all common genetic variability in and around the *NFE2L2* gene locus and investigated their influence on PD susceptibility using the large Queensland Parkinson’s Project case-control sample. To date, this is the largest focused candidate gene case-control study to examine the association of *NFE2L2* with PD, and the first to examine *NFE2L2* coding variants in PD patient cell lines. It is also the first to examine gene-environment interactions between *NFE2L2* SNPs and PD susceptibility.

Our association analysis reported two highly correlated *NFE2L2* SNPs that are significantly associated with a reduced risk of PD. One of these variants (rs2364725) has previously been reported to interact with increased exposure to particulate air pollutants, in a population of non-smoking myocardial infarction survivors, to prolong the QTc interval measured by an electrocardiogram [23]. This study retrospectively hypothesised that defence against oxidative stress is diminished in individuals with at least one minor allele. Paradoxically, in our study, the alternate allele of rs2364725 is protective for PD. However, no significant AAO, gene-environment interaction, gene expression, or cell viability differences for rs2364725 was observed in our study. It is currently unclear as to how this non-coding SNP in the 5’-region of the locus could impact mechanistically on NRF2 pathway function.

It is widely accepted that oxidative stress plays an important role in the onset of PD.

However, the mechanism is difficult to explore in isolation due to the multifactorial nature of the disease. Previous literature on PD risk factors has emphasised the importance of considering gene-environment interactions when exploring genetic factors involving biological pathways impacted upon by external exposures [24]. This is particularly important for *NFE2L2* variants, given the functional relevance of NRF2 in detoxification pathways.

To assess for possible gene-environment interactions between *NFE2L2* SNPs and xenobiotic exposures commonly associated with PD (smoking and pesticide exposure) we examined the joint effects of genotype and exposure on PD risk using logistic regression models. We found preliminary evidence that the alternate allele at rs2706110 may reduce PD risk in people regularly exposed to pesticides; however, this did not survive a correction for multiple hypotheses. In addition, baseline expression levels of *KEAP1* were found to be significantly lower in cell lines (n = 26) carrying the alternate allele of rs2706110 compared to cell lines (n = 26) containing the common allele. Reduced *KEAP1* expression may correspond with a reduction in the targeted post-translational degradation of NRF2 by KEAP1 and its subsequent stabilisation and translocation to the nucleus. We observed no differences in cell viability between common and alternate alleles for this SNP in rotenone treated cell lines. Further investigation is required to determine how the alternate allele of rs2706110, located in the non-coding 3’ region flanking *NFE2L2*, modulates *NFE2L2* transcriptional response to environmental pesticide exposure.

Our investigation into disease modifying genotypes identified a number of AAO-modifying SNPs; rs10183914 (*P*<0.001, 2.6 years/allele), rs6726395 (*P*<0.002, 1.9 years/allele), rs1806649 (*P*<0.001, 2.4 years/allele), and rs7557529 (*P* = 0.024, 1.6 years/allele). Larger haplotype windows containing these alternate alleles also exhibit the age-modifying effect of these SNPs. This may indicate that any age-modifying effect observed in larger haplotypes may be driven by these alternate alleles.

MAPs graphically portray how the differences in allele frequency, between cases and controls, move with the age of the participants in each group. In our analysis we were able to distinguish between risk factors and age-at-onset modifiers using this method. The results of our MAPs are in concordance with our association and AAO study.

There have been several isolated reports of genetic associations, particularly haplotypes around *NFE2L2* with PD. Our data has allowed a re-examination of some of these previous reports. None of the haplotypes generated from our tagging SNPs were associated with PD. In addition, none of the haplotypes associated with PD in previous studies were found to have a significant effect on disease susceptibility in our population (haplotypes VO1-7; Table 5).

For complete haplotype assessment, we investigated AAO effects in haplotypes generated from our promoter SNPs (P), tagging SNPs (T), replication haplotypes (VO), and combination haplotypes (promoter haplotypes in phases of the VO windows (VOP) and T windows (TP)) (S4 Table). We were able to replicate an ‘early-onset’ haplotype, P2 (TCT), which contains a previously identified ‘functional promoter’ SNP (rs6721961). Possession of the common allele, P3 (GCT), was conversely associated with a ‘disease-delaying’ AAO. This dichotomy of AAO was observed in all larger haplotype windows containing these alleles. Interestingly, rs6721961 was not found to independently modify AAO significantly (S2 Table). Haplotype P2 has previously been associated with significantly reduced *NFE2L2* promoter activity *in vitro* [19], and possession of this AAO-modifying allele may reflect changes in transcriptional activity. hONS cell lines containing the alternate allele were found to significantly overexpress *NQO1* compared to those containing the common allele (image C S4 Fig). Furthermore, a reduction in cell death was observed in rotenone treated cell lines, over time, containing the alternate allele (S5 Fig). Increased antioxidant capacity, in the form of *NQO1*, may correspond with an ability to mitigate xenobiotic insult. These findings contradict previous studies that suggest that the alternate allele at rs6721961 is associated with decreased transcriptional activity. No changes in *NFE2L2* and *KEAP1* expression were observed for this SNP. We did not examine this SNP in the larger case-control association study as this SNP was in the set selected for AAO investigation. It would be interesting to see whether this SNP is associated directly with disease risk, generally. Unfortunately, rs6721961 is not in LD with any of the examined SNPs in this study, therefore future experiments would be required to test this. It is most likely that our observation is a spurious finding, but further data is required to disprove this.

Replication haplotypes VO1 and VO2 were statistically associated with AAO changes of -1.4 years and 2.7 years, respectively (Fig 1 and S4 Table). Interestingly, haplotype VO2 exclusively contains all significant AAO-delaying SNPs (S2 Table). AAO effects were also observed for haplotypes VOP3 and VOP4 (*P =* 0.002, 2.963 years/allele and *P =* 0.003, -2.623 years/allele, respectively). In addition, the haplotypes TP3 (*P*<0.001, 3.307 years/allele) and TP4 (*P =* 0.003, -2.431 years/allele) were significantly associated with changes to AAO. All statistically significant associations survived a conservative Bonferroni correction for multiple comparisons.

A recent large-scale meta-analysis of all PD GWAS data failed to identify SNPs in *NFE2L2* that are associated with PD at a genome-wide significance level [25]. This suggests that independent main effects of *NFE2L2* genotype may not be strong genetic risk-factors for PD. However, these GWAS studies do not consider the potentially important interactions between environmental exposures and genotypes, which might exist in specific populations or in specific geographical contexts. Thus more focused studies of such interactions are warranted, especially considering that meta-analyses of *NFE2L2* SNPs from published data (as appears in PD Gene) suggest that there may, in certain populations, be some relationship between these genes and PD risk [25].

## Conclusion

Overall, we report a comprehensive genetic assessment of *NFE2L2* variants in PD. While we identified two rare *NFE2L2* coding variants from our hONS cell lines, it appears unlikely that they are pathogenic and are not likely the cause of NRF2-mediated pathway differences in PD hONS cell lines. The results we present here suggest that common *NFE2L2* variants may reduce PD susceptibility in certain conditions, such as regular exposure to pesticides. While our haplotype replication analysis did not confirm a previous disease association study, we did find significant AAO modification with a reported functional promoter SNP. Furthermore, we identified a number of SNPs associated with a significantly later AAO. These finding remained statistically significant following the stringent Bonferroni correction for multiple comparisons. Further functional analysis regarding the role of the disease-associated *NFE2L2* SNPs on NRF2 pathway expression and activity in hONS cell lines is warranted to elucidate their potential role in modulating PD susceptibility.

## Supporting Information

S1 Fig Linkage disequilibrium map of *NFE2L2* SNPs. *NFE2L2* gene schematic highlighting coding regions was derived from HapMap data (release 28, NCBI B36 assembly). Linkage disequilibrium map of *NFE2L2* tagging SNPs was generated from control subjects and visualised using Haplotype. Shading intensity of blocks indicates relative correlations between SNPs.
(TIF)

S2 Fig High resolution melting analysis of the *NFE2L2* coding regions. Normalized melting curves and difference plots for the (A) c.802G>A and (B) c.1230T>C variants. (C) DNA sequencing of the c.802G>A and c. 1230T>C variants.
(TIF)

S3 Fig Moving average plots of allele frequency against age. Plotted differences in allele frequency between cases (red boxes) and controls (blue diamonds) against binned age ranges (10 percentiles). Data shown is for significant AAO & association SNPs only.
(TIF)

S4 Fig Allele specific gene expression. Normalised gene expression for *NFE2L2*, *KEAP1*, and *NQO1* in hONS cell lines (total n = 52) for 11 tagging SNPs and 3 promoter SNPs. Major allele denotes common (wildtype) allele; Minor allele denotes possession of alternate allele. Error bars = standard deviation. * *P*<0.05.
(TIF)

S5 Fig Rotenone treated (50nM) cell viability assays. DMSO normalised cell number (CyQUANT) was measured after 1–5 days of rotenone treatment (50nM) of hONS cell lines (total n = 17) on an allele specific background. Major allele denotes common (wildtype) allele; Minor allele denotes possession of alternate allele. Error bars = standard deviation. * *P*<0.05.
(TIF)

S1 Table Primer design for high resolution melt analysis. *Lower case nucleotide sequences are intronic. ^Upper case nucleotide sequences are exonic.
(TIF)

S2 Table *NFE2L2* SNP AAO frequencies. ^a^ major/minor allele. ^b^ copy of alternate allele. ^c^ unstandardised B value in years. ^d^ 95% confidence intervals. ^e^ bonferroni corrected.
(DOCX)

S3 Table Gene-environment interactions. ^a^ major/minor allele. ^b^ odds ratio. ^c^ adjusted for age and gender. ^d^ ≥ 17 pack years (median). ^e^ lifetime exposure to pesticide >26 days.
(DOCX)

S4 Table Haplotype AAO frequencies. P = ‘Promotor haplotype’. VO = ‘von Otter haplotype’. T = ‘Tagging haplotype’. VOP = ‘von Otter plus promoter haplotype’. TP = ‘Tagging plus promotor haplotype.
(XLSX)

## References

[1] Lin MT, Beal MF. Mitochondrial dysfunction and oxidative stress in neurodegenerative diseases. Nature, 2006 443(7113):787–95. 17051205 10.1038/nature05292

[2] McGeer PL, Itagaki S, Boyes BE, McGeer EG. Reactive microglia are positive for HLA-DR in the substantia nigra of Parkinson's and Alzheimer's disease brains. Neurology, 1988 38(8):1285–91. 3399080 10.1212/wnl.38.8.1285

[3] Czlonkowska A, Kohutnicka M, Kurkowska-Jastrzebska I, Czlonkowski A. Microglial reaction in MPTP (1-methyl-4-phenyl-1,2,3,6-tetrahydropyridine) induced Parkinson's disease mice model. Neurodegeneration, 1996 5(2):137–43. 8819134 10.1006/neur.1996.0020

[4] Matigian N, Abrahamsen G, Sutharsan R, Cook AL, Vitale AM, Nouwens A, et al Disease-specific, neurosphere-derived cells as models for brain disorders. Dis Model Mech, 2010 3(11–12):785–98. 10.1242/dmm.004952 20699480

[5] Alfieri A, Srivastava S, Siow RC, Modo M, Fraser PA, Mann GE. Targeting the Nrf2-Keap1 antioxidant defence pathway for neurovascular protection in stroke. J Physiol, 2011 589(Pt 17):4125–36. 10.1113/jphysiol.2011.210294 21646410 PMC3180573

[6] Sykiotis GP, Habeos IG, Samuelson AV, Bohmann D. The role of the antioxidant and longevity-promoting Nrf2 pathway in metabolic regulation. Curr Opin Clin Nutr Metab Care, 2011 14(1):41–8. 10.1097/MCO.0b013e32834136f2 21102319 PMC3092636

[7] Kaidery NA, Banerjee R, Yang L, Smirnova NA, Hushpulian DM, Liby KT, et al Targeting Nrf2-mediated gene transcription by extremely potent synthetic triterpenoids attenuate dopaminergic neurotoxicity in the MPTP mouse model of Parkinson's disease. Antioxid Redox Signal, 2013 18(2): p. 139–57. 10.1089/ars.2011.4491 22746536 PMC3514006

[8] Lou H, Jing X, Wei X, Shi H, Ren D, Zhang X. Naringenin protects against 6-OHDA-induced neurotoxicity via activation of the Nrf2/ARE signaling pathway. Neuropharmacology, 2014 79:380–8. 10.1016/j.neuropharm.2013.11.026 24333330

[9] Song J, Kang SM, Lee WT, Park KA, Lee KM, Lee JE. Glutathione protects brain endothelial cells from hydrogen peroxide-induced oxidative stress by increasing nrf2 expression. Exp Neurobiol, 2014 23(1):93–103. 10.5607/en.2014.23.1.93 24737944 PMC3984961

[10] Suh JH, Shenvi SV, Dixon BM, Liu H, Jaiswal AK, Liu RM, et al Decline in transcriptional activity of Nrf2 causes age-related loss of glutathione synthesis, which is reversible with lipoic acid. Proc Natl Acad Sci U S A, 2004 101(10):3381–6. 14985508 10.1073/pnas.0400282101 PMC373470

[11] Alam ZI, Daniel SE, Lees AJ, Marsden DC, Jenner P, Halliwell B. A generalised increase in protein carbonyls in the brain in Parkinson's but not incidental Lewy body disease. J Neurochem, 1997 69(3):1326–9. 9282961 10.1046/j.1471-4159.1997.69031326.x

[12] Settivari R, VanDuyn N, LeVora J, Nass R. The Nrf2/SKN-1-dependent glutathione S-transferase pi homologue GST-1 inhibits dopamine neuron degeneration in a Caenorhabditis elegans model of manganism. Neurotoxicology, 2013 38:51–60. 10.1016/j.neuro.2013.05.014 23721876 PMC3773487

[13] Innamorato NG, Jazwa A, Rojo AI, Garcia C, Fernandez-Ruiz J, Grochot-Przeczek A, et al Different susceptibility to the Parkinson's toxin MPTP in mice lacking the redox master regulator Nrf2 or its target gene heme oxygenase-1. PLoS One, 2010 5(7): e11838 10.1371/journal.pone.0011838 20676377 PMC2911386

[14] Chien WL, Lee TR, Hung SY, Kang KH, Lee MJ, Fu WM. Impairment of oxidative stress-induced heme oxygenase-1 expression by the defect of Parkinson-related gene of PINK1. J Neurochem, 2011 117(4):643–53. 10.1111/j.1471-4159.2011.07229.x 21366594

[15] Xiao H, Lv F, Xu W, Zhang L, Jing P, Cao X. Deprenyl prevents MPP(+)-induced oxidative damage in PC12 cells by the upregulation of Nrf2-mediated NQO1 expression through the activation of PI3K/Akt and Erk. Toxicology, 2011 290(2–3):286–94. 10.1016/j.tox.2011.10.016 22019741

[16] Cook AL, Vitale AM, Ravishankar S, Matigian N, Sutherland GT, Shan J, et al NRF2 activation restores disease related metabolic deficiencies in olfactory neurosphere-derived cells from patients with sporadic Parkinson's disease. PLoS One, 2011 6(7):e21907 10.1371/journal.pone.0021907 21747966 PMC3128624

[17] von Otter M, Landgren S, Nilsson S, Celojevic D, Bergstrom P, Hakansson A, et al Association of Nrf2-encoding NFE2L2 haplotypes with Parkinson's disease. BMC Med Genet, 2010 11:36 10.1186/1471-2350-11-36 20196834 PMC2843602

[18] Chen YC, Wu YR, Wu YC, Lee-Chen GJ, Chen CM. Genetic analysis of NFE2L2 promoter variation in Taiwanese Parkinson's disease. Parkinsonism Relat Disord, 2013 19(2):247–50. 10.1016/j.parkreldis.2012.10.018 23176750

[19] Marzec JM, Christie JD, Reddy SP, Jedlicka AE, Vuong H, Lanken PN, et al Functional polymorphisms in the transcription factor NRF2 in humans increase the risk of acute lung injury. FASEB J, 2007 21(9):2237–46. 17384144 10.1096/fj.06-7759com

[20] Sutherland GT, Halliday GM, Silburn PA, Mastaglia FL, Rowe DB, Boyle RS, et al Do polymorphisms in the familial Parkinsonism genes contribute to risk for sporadic Parkinson's disease? Mov Disord, 2009 24(6):833–8. 10.1002/mds.22214 19224617

[21] Gartner CE, Battistutta D, Dunne MP, Silburn PA, Mellick GD. Test-retest repeatability of self-reported environmental exposures in parkinson’s disease cases and healthy controls. Parkinsonism & Related Disorders, 2005 11(5):287–295 15994111 10.1016/j.parkreldis.2005.04.002

[22] Payami H, Kay DM, Zabetian CP, Schellenberg GD, Factor SA, McCulloch CC. Visualizing disease associations: graphic analysis of frequency distributions as a function of age uing moving average plots (MAP) with application to Alzheimer’s and Parkinson’s disease. Genet. Epidemiol., 2010 34(1):92–9. 10.1002/gepi.20439 19582778 PMC2796703

[23] Hampel R, Schneider A. Bruske I, Zareba W, Cyrys J, Ruckerl R, et al Altered cardiac repolarization in association with air pollution and air temperature among myocardial infarction survivors. Environ Health Perspect, 2010 118(12):1755–61. 10.1289/ehp.1001995 20846924 PMC3002196

[24] Mellick GD. CYP450, genetics and Parkinson's disease: gene x environment interactions hold the key. J Neural Transm Suppl, 2006(70):159–65. 17017524 10.1007/978-3-211-45295-0_25

[25] Nalls MA, Pankratz N, Lill CM, Do CB, Hernandez DG, Saad M, et al Large-scale meta-analysis of genome-wide association data indentifies six new risk loci for Parkinson’s disease. Nat Genet, 2014 46(9):989–93. 10.1038/ng.3043 25064009 PMC4146673

