# Supplementary figures and images for: Comprehensive Assessment of Genetic Sequence Variants in the Antioxidant ‘Master Regulator’ Nrf2 in Idiopathic Parkinson’s Disease
Source: PLoS One. 2015 May 26;10(5):e0128030. doi: 10.1371/journal.pone.0128030 (PMC4444110; doi:10.1371/journal.pone.0128030)

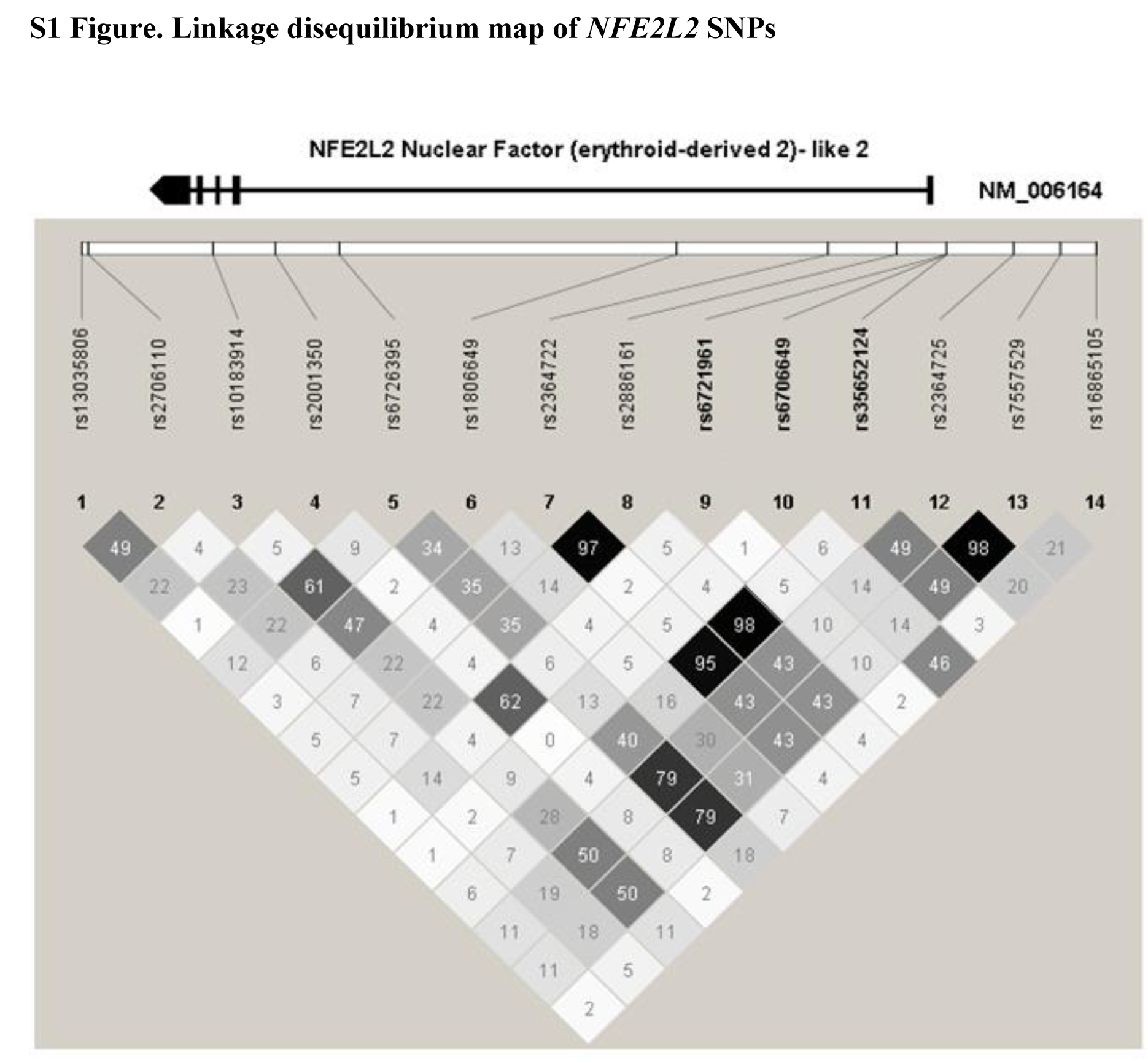

Supplement: S1 Fig — NFE2L2 gene schematic highlighting coding regions was derived from HapMap data (release 28, NCBI B36 assembly). Linkage disequilibrium map of NFE2L2 tagging SNPs was generated from control subjects and visualised using Haplotype. Shading intensity of blocks indicates relative correlations between SNPs. (TIF) [file pone.0128030.s001.tif]

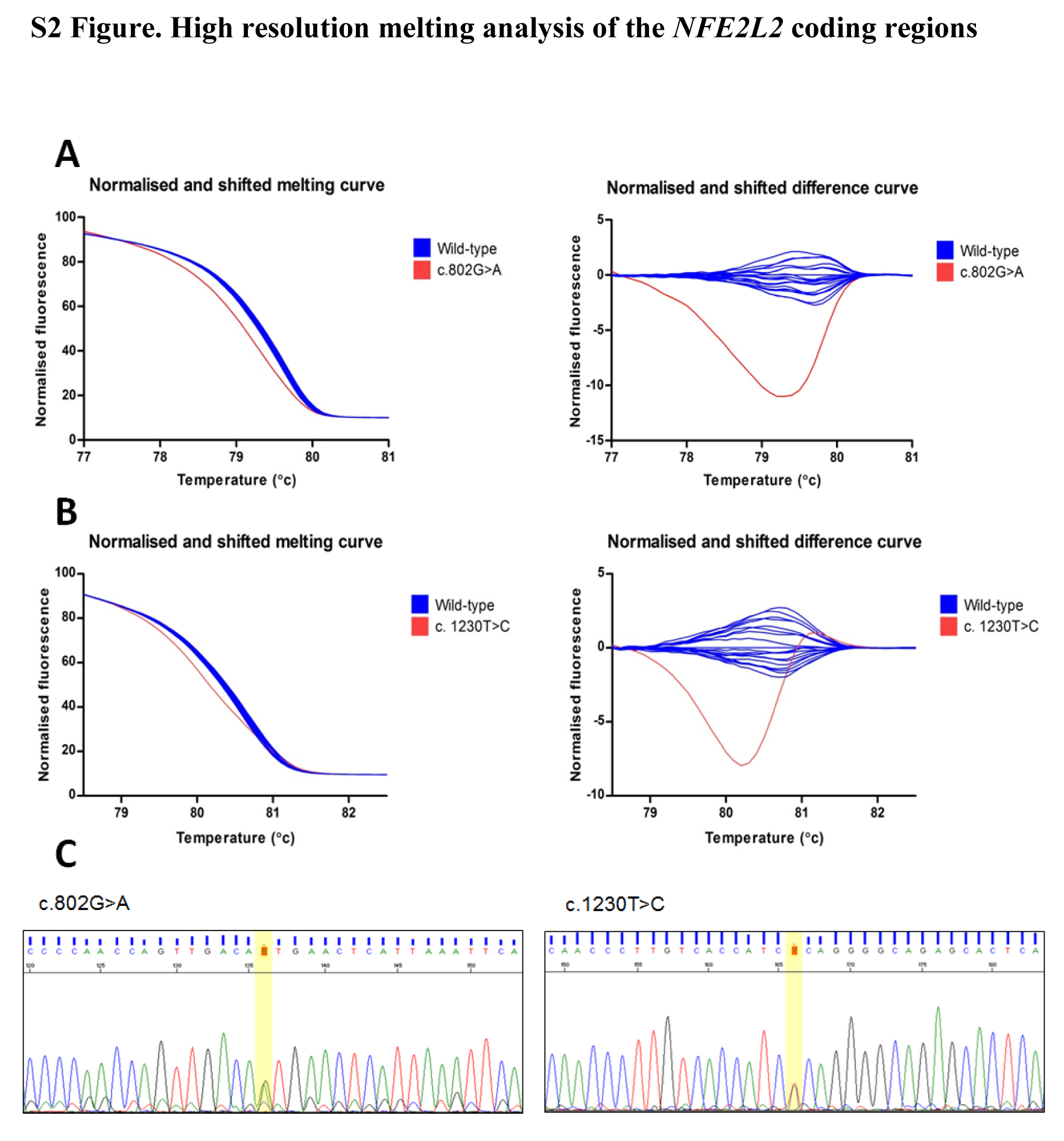

Supplement: S2 Fig — Normalized melting curves and difference plots for the (A) c.802G>A and (B) c.1230T>C variants. (C) DNA sequencing of the c.802G>A and c. 1230T>C variants. (TIF) [file pone.0128030.s002.tif]

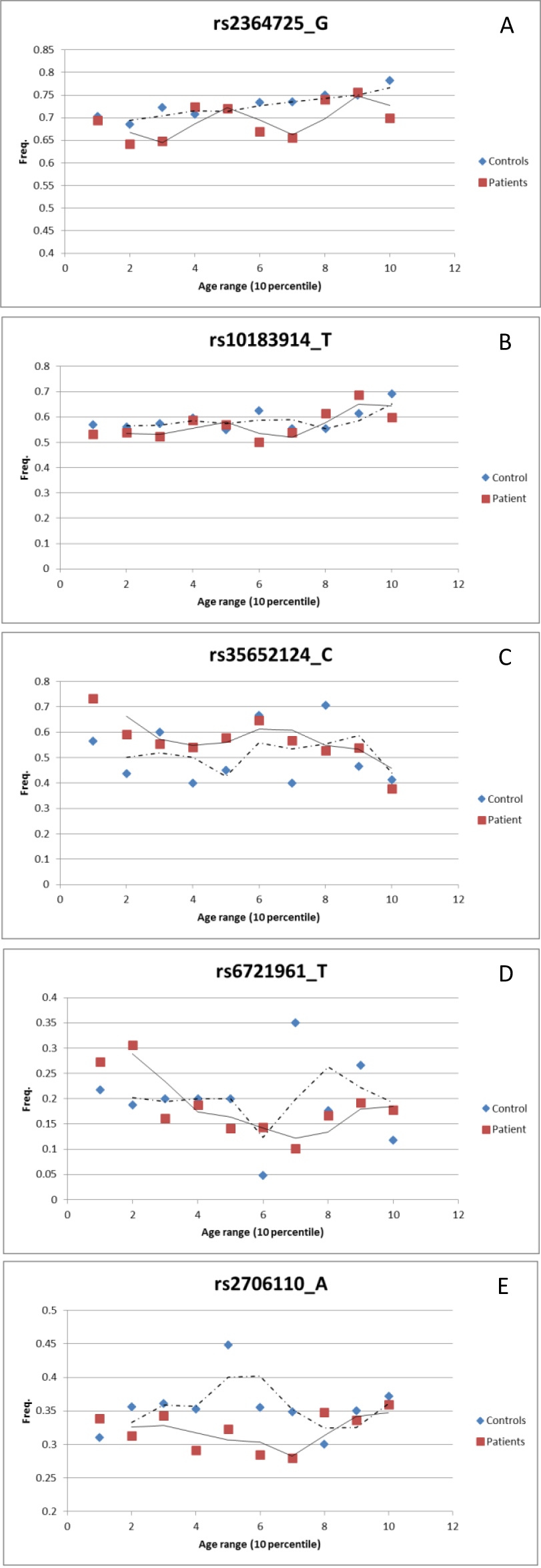

Supplement: S3 Fig — Plotted differences in allele frequency between cases (red boxes) and controls (blue diamonds) against binned age ranges (10 percentiles). Data shown is for significant AAO & association SNPs only. (TIF) [file pone.0128030.s003.tif]

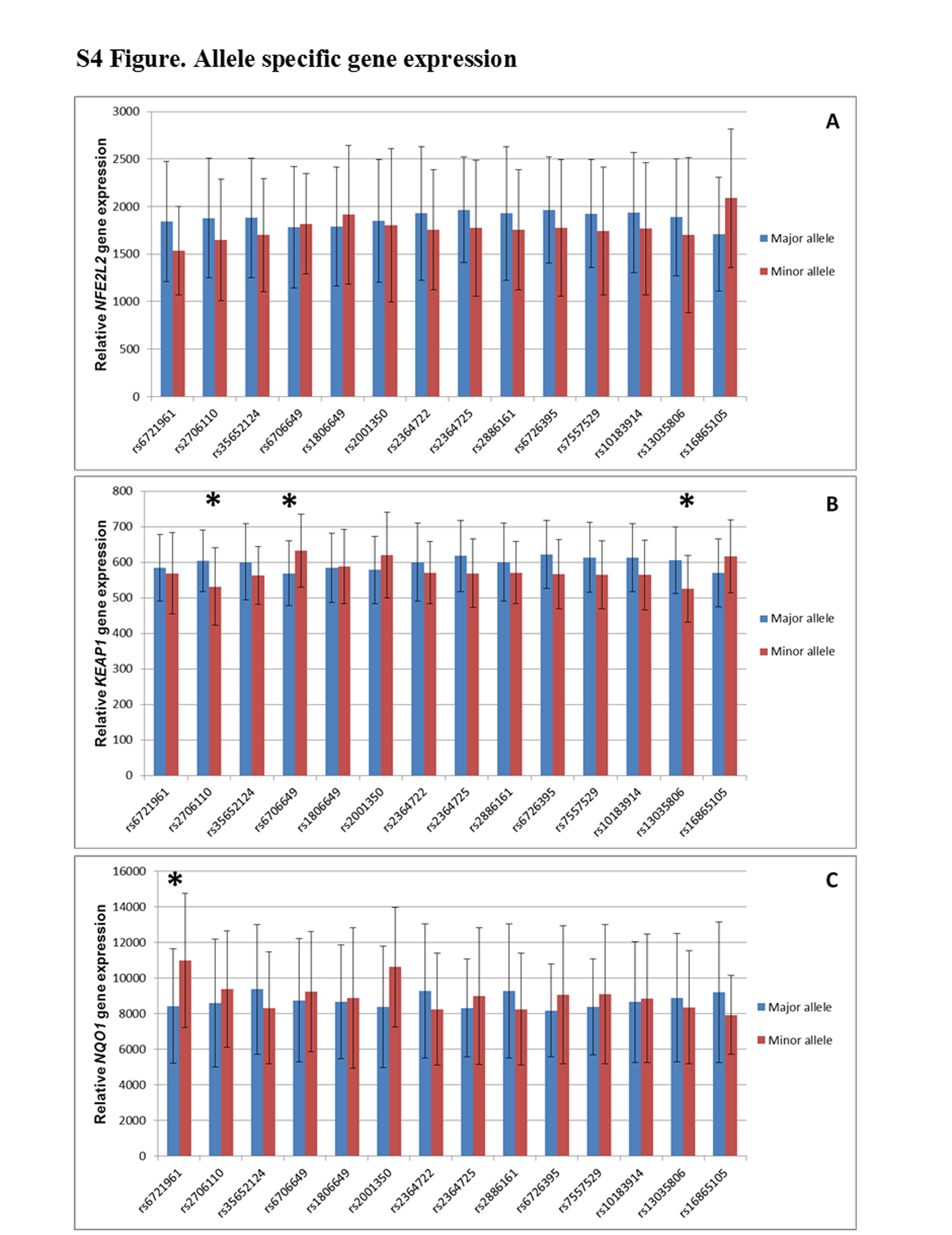

Supplement: S4 Fig — Normalised gene expression for NFE2L2, KEAP1, and NQO1 in hONS cell lines (total n = 52) for 11 tagging SNPs and 3 promoter SNPs. Major allele denotes common (wildtype) allele; Minor allele denotes possession of alternate allele. Error bars = standard deviation. * P<0.05. (TIF) [file pone.0128030.s004.tif]

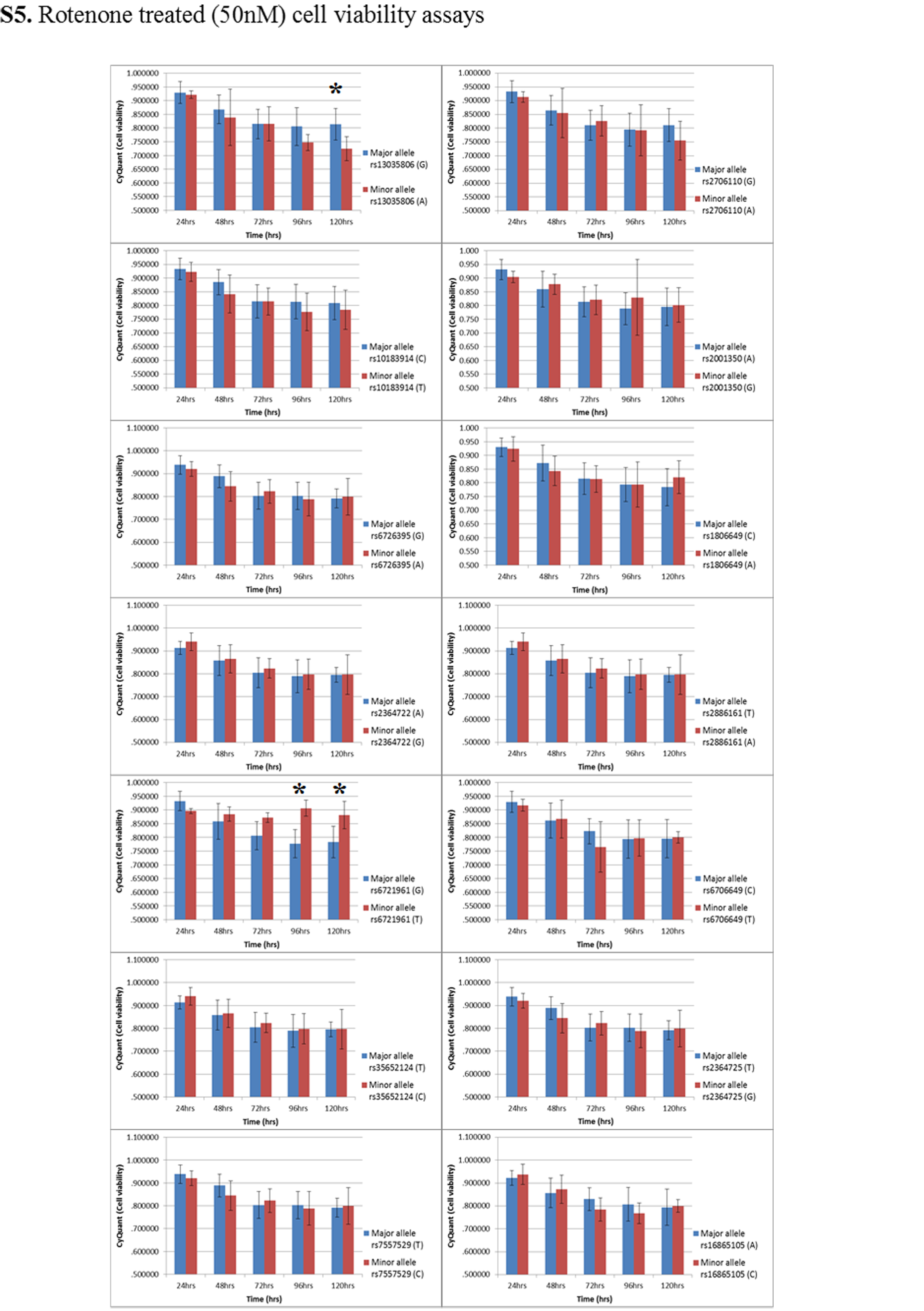

Supplement: S5 Fig — DMSO normalised cell number (CyQUANT) was measured after 1–5 days of rotenone treatment (50nM) of hONS cell lines (total n = 17) on an allele specific background. Major allele denotes common (wildtype) allele; Minor allele denotes possession of alternate allele. Error bars = standard deviation. * P<0.05. (TIF) [file pone.0128030.s005.tif]

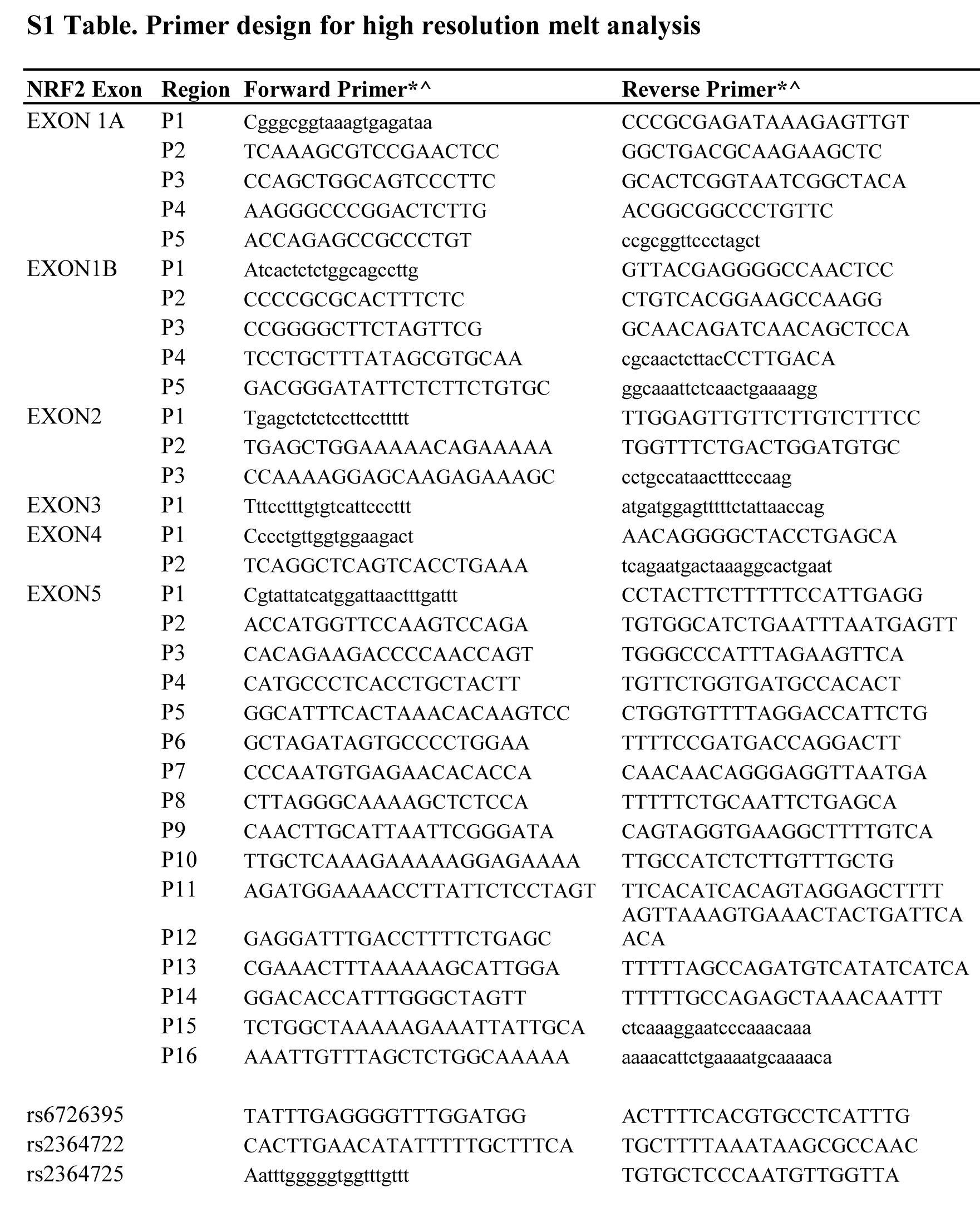

Supplement: S1 Table — *Lower case nucleotide sequences are intronic. ^Upper case nucleotide sequences are exonic. (TIF) [file pone.0128030.s006.tif]
