# Supplementary material for: Comprehensive Assessment of Genetic Sequence Variants in the Antioxidant ‘Master Regulator’ Nrf2 in Idiopathic Parkinson’s Disease
Source: PLoS One. 2015 May 26;10(5):e0128030. doi: 10.1371/journal.pone.0128030 (PMC4444110; doi:10.1371/journal.pone.0128030)
Supplement: S2 Table — a major/minor allele. b copy of alternate allele. c unstandardised B value in years. d 95% confidence intervals. e bonferroni corrected. (DOCX) [file pone.0128030.s007.docx]

**S2 Table. *NFE2L2* SNP AAO frequencies**

|  |  | No copies **^b^** | | 1 copy **^b^** | | 2 copies **^b^** | |  |  | |
| --- | --- | --- | --- | --- | --- | --- | --- | --- | --- | --- |
| SNP | Alleles **^a^** | N | Mean±SD | N | Mean±SD | N | Mean±SD | B**^c^** (95% CI)**^d^** | *P* **^e^** | |
| rs13035806 | G/A | 882 | 58.963 ± 11.510 | 193 | 60.694 ± 11.232 | 16 | 60.813 ± 9.840 | 1.487 (-0.062, 3.036) | 1 | |
| rs2706110 | G/A | 743 | 59.211 ± 11.193 | 311 | 59.653 ± 11.983 | 36 | 58.889 ± 12.565 | 0.205 (-1.052, 1.463) | 1 | |
| rs10183914 | C/T | 471 | 57.590 ± 12.136 | 494 | 59.937 ± 10.840 | 129 | 63.101 ± 10.204 | 2.625 (1.623, 3.628) | <0.001 | |
| rs2001350 | A/G | 910 | 59.555 ± 11.175 | 176 | 58.000 ± 12.685 | 8 | 58.875 ± 16.444 | -1.330 (-3.035, 0.376) | 1 | |
| rs6726395 | G/A | 345 | 57.670 ± 11.716 | 549 | 59.525 ± 11.217 | 199 | 61.538 ± 11.410 | 1.929 (0.952, 2.907) | 0.002 | |
| rs1806649 | C/T | 617 | 58.164 ± 11.997 | 410 | 60.354 ± 10.633 | 67 | 63.313 ± 10.061 | 2.389 (1.282, 3.496) | <0.001 | |
| rs2364722 | A/G | 483 | 60.464 ± 11.104 | 478 | 58.404 ± 12.063 | 126 | 58.270 ± 10.349 | -1.430 (-2.445, -0.416) | 0.081 | |
| rs2886161 | T/C | 489 | 60.542 ± 11.116 | 476 | 58.311 ± 12.007 | 129 | 58.240 ± 10.376 | -1.514 (-2.519, -0.509) | 0.045 | |
| rs6721961 | G/T | 378 | 59.840 ± 11.092 | 77 | 56.080 ± 13.041 | 6 | 61.0 ± 15.073 | -2.636 (-5.108, -0.164) | 0.514 | |
| rs6706649 | C/T | 347 | 59.110 ± 11.516 | 106 | 59.510 ± 12.018 | 9 | 58.110 ± 8.810 | 0.119 (-2.070, 2.307) | 1 | |
| rs35652124 | T/C | 202 | 61.080 ± 11.004 | 211 | 57.790 ± 12.170 | 49 | 57.370 ± 10.161 | -2.234 (-3.947, -0.750) | 0.057 | |
| rs2364725 | T/G | 314 | 57.844 ± 11.647 | 558 | 59.523 ± 11.422 | 216 | 60.667 ± 11.029 | 1.444 (0.463, 2.424) | 0.055 |  |
| rs7557529 | T/C | 308 | 57.883 ± 11.489 | 562 | 59.365 ± 11.528 | 223 | 61.022 ± 11.091 | 1.566 (0.587, 2.546) | 0.024 |  |
| rs16865105 | A/C | 732 | 59.429 ± 11.385 | 312 | 58.978 ± 11.901 | 49 | 59.653 ± 11.475 | -0.191 (-1.388, 1.006) | 1 |  |
