# Supplementary material for: Comprehensive Assessment of Genetic Sequence Variants in the Antioxidant ‘Master Regulator’ Nrf2 in Idiopathic Parkinson’s Disease
Source: PLoS One. 2015 May 26;10(5):e0128030. doi: 10.1371/journal.pone.0128030 (PMC4444110; doi:10.1371/journal.pone.0128030)
Supplement: S3 Table — a major/minor allele. b odds ratio. c adjusted for age and gender. d ≥ 17 pack years (median). e lifetime exposure to pesticide >26 days. (DOCX) [file pone.0128030.s008.docx]

**S3.** Gene-environment interactions

| **SNP** | **Alleles ^a^** | **OR ^b^ (95% CI)** | ***P* ^c^** | ***Bonferroni***  ***Corrected P*** | |
| --- | --- | --- | --- | --- | --- |
| **Main Effects (without interaction)** | | | |  |  |
| rs2364725 | T/T | ref | - | |  |
|  | T/G | 0.895 (0.741-1.082) | 0.252 | | 1 |
|  | G/G | 0.744 (0.592-0.935) | 0.011 | | 0.152 |
| rs2364725 (additive model) | | 0.849 (0.760-0.948) | 0.004 | | 0.054 |
| Smoking ^d^ |  | 0.499 (0.402-0.618) | <0.005 | | <0.005 |
| Pesticide exposure ^e^ | | 1.562 (1.228-1.988) | <0.005 | | <0.005 |
| **Final Interaction model (additive genetic model)** | | | | |  |
| rs2364725 | | 0.881 (0.766-1.013) | 0.074 | | 1 |
| Smoking | | 0.671 (0.482-0.934) | <0.005 | | 0.002 |
| Pesticide | | 2.031 (1.355-3.043) | <0.005 | | 0.008 |
| Smoking ^d^ * rs2364725 | | 0.976 (0.730-1.304) | 0.867 | | 1 |
| Pesticide ^e^ * rs2364725 | | 0.765 (0.551-1.106) | 0.109 | | 1 |

| **SNP** | **Alleles ^a^** | **OR ^b^ (95% CI)** | ***P* ^c^** | ***Bonferroni***  ***Corrected P*** | |
| --- | --- | --- | --- | --- | --- |
| **Main Effects (without interaction)** | | | |  |  |
| rs6721961 | G/G | ref | - | |  |
|  | G/T | 0.851 (0.541-1.337) | 0.484 | | 1 |
|  | T/T | 1.174 (0.230-6.002) | 0.847 | | 1 |
| rs6721961 (additive model) | | 0.902 (0.605-1.344) | 0.611 | | 1 |
| Smoking ^d^ |  | 0.494 (0.321-0.761) | <0.005 | | 0.019 |
| Pesticide exposure ^e^ | | 1.009 (0.612-1.664) | 0.971 | | 1 |
| **Final Interaction model (additive genetic model)** | | | | |  |
| rs6721961 | | 0.921 (0.573-1.482) | 0.735 | | 1 |
| Smoking | | 0.500 (0.315-0.796) | <0.005 | | 0.048 |
| Pesticide | | 1.020 (0.586-1.776) | 0.944 | | 1 |
| Smoking ^d^ * rs6721961 | | 0.939 (0.362-2.439) | 0.897 | | 1 |
| Pesticide ^e^ * rs6721961 | | 0.959 (0.309-2.977) | 0.942 | | 1 |

| **SNP** | **Alleles ^a^** | **OR ^b^ (95% CI)** | ***P* ^c^** | ***Bonferroni***  ***Corrected P*** | |
| --- | --- | --- | --- | --- | --- |
| **Main Effects (without interaction)** | | | |  |  |
| rs10183914 | C/C | ref | - | |  |
|  | C/T | 0.851 (0.541-1.337) | 0.484 | | 1 |
|  | T/T | 1.174 (0.230-6.002) | 0.847 | | 1 |
| rs10183914 (additive model) | | 0.911 (0.811-1.023) | 0.114 | | 1 |
| Smoking ^d^ |  | 0.515 (0.416-0.637) | <0.005 | | <0.005 |
| Pesticide exposure ^e^ | | 1.543 (1.210-1.967) | <0.005 | | 0.006 |
| **Final Interaction model (additive genetic model)** | | | | |  |
| rs10183914 | | 0.957 (0.827-1.107) | 0.550 | | 1 |
| Smoking | | 0.500 (0.315-0.796) | <0.005 | | <0.005 |
| Pesticide | | 1.020 (0.586-1.776) | <0.005 | | 0.015 |
| Smoking ^d^ * rs10183914 | | 0.939 (0.362-2.439) | 0.897 | | 1 |
| Pesticide ^e^ * rs10183914 | | 1.770 (1.157-2.492) | 0.001 | | 1 |

| **SNP** | **Alleles ^a^** | **OR ^b^ (95% CI)** | ***P* ^c^** | ***Bonferroni***  ***Corrected P*** | |
| --- | --- | --- | --- | --- | --- |
| **Main Effects (without interaction)** | | | |  |  |
| rs35652124 | T/T | ref | - | |  |
|  | T/C | 1.326 (0.854-2.060) | 0.209 | | 1 |
|  | C/C | 0.819 (0.416-1.614) | 0.564 | | 1 |
| rs35652124 (additive model) | | 1.167 (0.897-1.519) | 0.250 | | 1 |
| Smoking ^d^ |  | 0.487 (0.316-0.750) | <0.005 | | 0.015 |
| Pesticide exposure ^e^ | | 1.039 (0.630-1.715) | 0.880 | | 1 |
| **Final Interaction model (additive genetic model)** | | | | |  |
| rs35652124 | | 1.030 (0.751-1.411) | 0.855 | | 1 |
| Smoking | | 0.404(0.225-0.724) | <0.005 | | 0.032 |
| Pesticide | | 0.841 (0.437-1.617) | 0.603 | | 1 |
| Smoking ^d^ * rs35652124 | | 1.351 (0.736-2.480) | 0.331 | | 1 |
| Pesticide ^e^ * rs35652124 | | 1.535 (0.668-3.528) | 0.313 | | 1 |
